# Exercise-Induced Benefits for Alzheimer’s Disease by Stimulating Mitophagy and Improving Mitochondrial Function

**DOI:** 10.3389/fnagi.2021.755665

**Published:** 2021-10-01

**Authors:** Jiling Liang, Cenyi Wang, Hu Zhang, Jielun Huang, Juying Xie, Ning Chen

**Affiliations:** ^1^Tianjiu Research and Development Center for Exercise Nutrition and Foods, Hubei Key Laboratory of Exercise Training and Monitoring, College of Health Science, Wuhan Sports University, Wuhan, China; ^2^School of Physical Education and Sports Science, Soochow University, Suzhou, China; ^3^Affiliated Hospital of Xiangnan University, Chenzhou, China

**Keywords:** Alzheimer’s disease, exercise, mitophagy, mitochondrial quality control, oxidative stress

## Abstract

Neurons are highly specialized post-mitotic cells that are inherently dependent on mitochondria due to their higher bioenergetic demand. Mitochondrial dysfunction is closely associated with a variety of aging-related neurological disorders, such as Alzheimer’s disease (AD), and the accumulation of dysfunctional and superfluous mitochondria has been reported as an early stage that significantly facilitates the progression of AD. Mitochondrial damage causes bioenergetic deficiency, intracellular calcium imbalance and oxidative stress, thereby aggravating β-amyloid (Aβ) accumulation and Tau hyperphosphorylation, and further leading to cognitive decline and memory loss. Although there is an intricate parallel relationship between mitochondrial dysfunction and AD, their triggering factors, such as Aβ aggregation and hyperphosphorylated Tau protein and action time, are still unclear. Moreover, many studies have confirmed abnormal mitochondrial biosynthesis, dynamics and functions will present once the mitochondrial quality control is impaired, thus leading to aggravated AD pathological changes. Accumulating evidence shows beneficial effects of appropriate exercise on improved mitophagy and mitochondrial function to promote mitochondrial plasticity, reduce oxidative stress, enhance cognitive capacity and reduce the risks of cognitive impairment and dementia in later life. Therefore, stimulating mitophagy and optimizing mitochondrial function through exercise may forestall the neurodegenerative process of AD.

## Introduction

With the aggravation of population aging, dementia has become the third killer after cancers and cardiovascular diseases, and it is one of the most challenging diseases for healthcare professionals today. According to the statistics from the United States as of 2021, approximately 6.2 million Americans at the age of 65 and older live with Alzheimer’s dementia today; this figure could grow to 13.8 million by 2060 (Alzheimer’s Association, 2021). Alzheimer’s disease (AD), a complicated, multifactorial, heterogeneous neurodegenerative disorder, is the most common form of dementia, characterized by a progressive loss of memory and cognitive capacity. Although the incidence of AD continues to rise, there are currently no effective disease-modifying drugs for the treatment of AD.

At present, the pathogenesis of AD has not been clearly clarified, and the common features for the clinical diagnosis of AD are senile plaques (SPs) from extracellular β-amyloid (Aβ) deposits and intraneuronal neurofibrillary tangles (NFTs) due to the aggregated and hyperphosphorylated Tau protein (Khan et al., 2020). Given the background, Hardy and Higgins first proposed the amyloid cascade hypothesis that Aβ protein aggregation is the initiating factor for the pathological damage during the AD process. The above hypothesis believes the deposition of Aβ in the brain is the initial and central link in the pathological changes of AD (Hardy and Higgins, 1992), which induces a series of pathological processes such as Aβ plaques, Tau phosphorylation, NFTs and neuronal death. These pathological processes increase the deposition of Aβ, thus forming cascade amplification and ultimately leading to declined cognitive capacity (Liu et al., 2019). The amyloid cascade hypothesis has been regarded as the major pathogenic concept in AD studies in the past few decades. However, recent clinical studies based on amyloid cascade hypothesis have been challenged by disappointed results (Sperling et al., 2011; Selfridge et al., 2013), and this hypothesis is far from explaining pathological mechanisms of AD, but its combination with other hypotheses may be the future trend of exploring AD treatment.

More and more evidences show that the impairment of mitochondrial function is the major pathological factor of aging-related neurodegenerative diseases (NDs) (Stanga et al., 2020; Johnson et al., 2021). It is worth noting that although bioenergetic defects and related oxidative stress are the leading cause of NDs, the evidence of impaired mitochondrial dynamics, biogenesis, and autophagy has recently demonstrated a causative link between alteration in mitochondrial function and NDs (Cabezas-Opazo et al., 2015). Therefore, a wide range of mechanisms for maintaining mitochondria at an appropriate functional status is essential to prevent diseases related to mitochondrial dysfunction and cell death (Wu et al., 2019). In this case, targeting mitochondrial dysfunction may be a potential therapeutic strategy to delay or prevent the early neurodegenerative process of AD and attenuate neuronal death.

Aging is the primary risk factor for the development and progression of AD, and physical inactivity has been recognized as an important account for increased morbidity and mortality of AD patients (Norton et al., 2014). Meanwhile, mitochondrial metabolism in brain seems to be highly modulated by exercise. Exercise has been confirmed as an ideal non-pharmacological therapy to promote cognitive capacity, improve mitochondrial dysfunction, and effectively delay and rescue the declined cognitive functions such as memory in the elderly (Cass, 2017; Luo et al., 2017; Zhao et al., 2021). This article summarizes oxidative stress, biogenesis, dynamics and mitophagy of mitochondria involved in the aging process, the correlation between AD due to dysfunctional mitochondria and corresponding exercise interventions, and the underlying mechanisms of corresponding factors for affecting these processes.

## Mitochondrial Cascade Hypothesis

Many experimental evidences demonstrate that the late-stage fibrillar deposits of phosphorylated Tau and the accumulation of Aβ represent the characteristic neuropathological features of AD (Swerdlow, 2012). However, the alterations for causing Aβ accumulation and hyperphosphorylated Tau in cellular homeostasis are unclear. In this case, multiple evidences have documented simple plaque development alone does not cause neurotoxicity. In addition to the deposition of plaques and tangles, other processes can seriously affect mitochondrial functions of the brain with AD (Perez Ortiz and Swerdlow, 2019). According to previous literature reports, the level of mitochondrial DNA (mtDNA) in neurons presents a declining trend before the formation of NFTs (Mao and Reddy, 2011), and the activity of tricarboxylic acid (TCA) cycle enzymes is significantly reduced (Bubber et al., 2005), accompanied by the reduction of glucose metabolism in the brain (Azari et al., 1993). Thus, the mitochondrial function of brain cells is affected by Aβ and Tau pathology (Eckert et al., 2011), and the dysfunctional or damaged mitochondria represent the critical early neuropathological signs of AD (Gibson and Shi, 2010).

**FIGURE 1:**
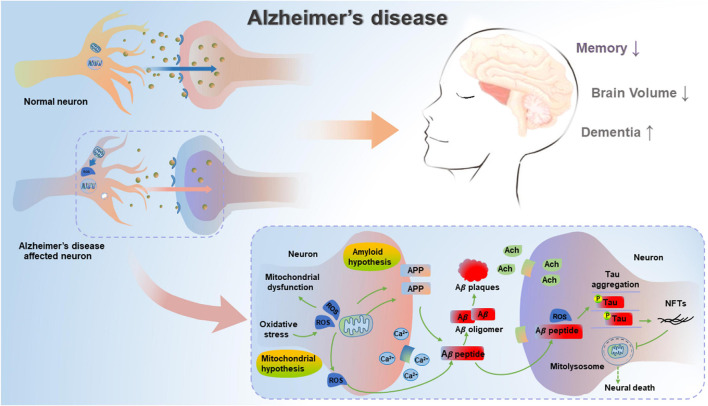
Mitochondrial cascade hypothesis and amyloid cascade hypothesis in AD. In unaffected neurons, healthy mitochondria are distributed through the neuron. In AD-affected neurons, mitochondrial dysfunction causes deficient energy metabolism and increased oxidative stress, thereby leading to increased amyloidogenic process of APP and the aggregation of hyperphosphorylated Tau. Pathogenic Aβ and hyperphosphorylated Tau can result in impaired mitophagy, subsequent increase in damaged or dysfunctional mitochondria, and blockage in mitolysosome, thereby leading to neural death in AD.

Subsequently, Swerdlow and Khan have proposed a mitochondrial cascade hypothesis that genetic factors determine the basal mitochondrial function of individuals, and the speed of aging-induced mitochondrial change is determined by environmental factors (Swerdlow and Khan, 2004). Therefore, the accumulation of damaged mitochondria could result in both neuropathological changes and corresponding symptoms of AD. In the case of this hypothesis, aging represents a leading risk factor for the development of sporadic AD (SAD), and the accumulation of Aβ is the aging-induced result, not the cause of neuropathological evolution (Swerdlow et al., 2010). This hypothesis is confirmed by the formation of SP due to Aβ deposits, deficient energy metabolism and increased oxidative stress (Lejri et al., 2019), as well as aberrant mitochondrial morphology and functions (Oliver and Reddy, 2019; Pradeepkiran and Reddy, 2020), indicating that mitochondrial function can affect the formation of amyloid β precursor protein (APP) and the accumulation of Aβ in AD. The mitochondrial cascade hypothesis is a supplement to the amyloid cascade hypothesis, suggesting the important role of the functional status of mitochondria in the production, modification and accumulation of Aβ and Tau, as well as the formation of oligomers (Swerdlow, 2018). In addition, another report has also described the accumulated Aβ- and pathogenic Tau-driven mitochondrial dysfunction (Mossmann et al., 2014). Whether mitochondrial dysfunction can lead to AD or corresponding pathologies can subsequently cause mitochondrial dysfunction is a well-debated topic. Hence, mitochondrial dysfunction can be an upstream inducer of Aβ and hyperphosphorylated Tau, while Aβ and hyperphosphorylated Tau can further exacerbate mitochondrial dysfunction, thereby resulting in a vicious cycle reaction in AD (Figure 1).

## Mitochondrial Dysfunction and Its Correlation With Alzheimer’s Disease

**FIGURE 2:**
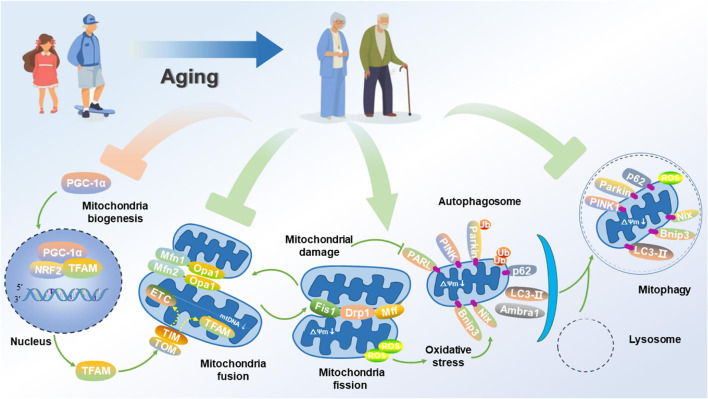
Impairment of mitochondrial function and mitophagy in AD. Aged neuron displays attenuated PGC-1α signaling for mitochondrial biogenesis, disrupted mitochondrial fusion and fission, and suppressed mitophagy flux, thus promoting a fragmented network of organelles. Consequently, these events activate PGC-1α, thus allowing its translocation to the nucleus where it co-activates NRF2 to up-regulate the expression of TFAM. Aging causes some areas of the mitochondrial network to be dysfunctional. Mitochondrial fusion proteins such as Mfn1/2 and Opa1 for promoting the fusion of inner and outer membranes are down-regulated. Alternatively, damaged organelles undergo fission, mediated by the interaction of proteins such as Fis1, Drp1, and Mfn1/2 to constrict and remove the organelles, thus allowing for their clearance via mitophagy. Once separated, damaged mitochondria with a low ΔΨm accumulation of PINK1 on their outer membrane. PINK1 recruits the Parkin, which subsequently ubiquitinates outer membrane proteins to flag the organelle for removal via mitophagy. The adapter p62 binds to the ubiquitin on the tagged cargo and LC3-II embedded in the phagophore membrane for promoting the formation of the autophagosome, thereby fusing with the lysosome to degrade the damaged or dysfunctional mitochondria.

As the critical cellular organelles, mitochondria play a dominant role in neurophysiological function, thus supporting cell survival by integrating cell respiration, energy metabolism and Ca^2+^ balance. Under physiological conditions, the fusion of healthy and damaged mitochondria can dilute damaged materials into the healthy mitochondrial network to avoid the build-up of dysfunctional mitochondria. After mitochondrial fission, the dysfunctional or damaged parts of the mitochondria are isolated and eventually cleared by mitochondrial phagocytosis, thus effectively maintaining mitochondrial quantity and quality Youle and van der Bliek (2012). Since diseases associated with mitochondrial dysfunction were first discovered in (Luft et al., 1962), the roles of mitochondria in health, disease and aging have now been extensively studied and confirmed. In this regard, a large amount of data confirms that mitochondrial dysfunction is a causative factor in the development of AD. Based on the mitochondrial cascade hypothesis, aging directly accelerates the damage of mitochondria in neurons of the brain, triggers an increase in mitochondrial oxidative stress, weakens mitochondrial biogenesis, thereby leading to the imbalance of mitochondrial dynamics, suppressing mitophagy, disrupting the quality control of mitochondria, and aggravating the pathological processes of AD (Figure 2).

### Oxidative Stress in Alzheimer’s Disease

The function of mitochondria to regulate healthy neurons is mainly affected by aging and pathological status. Mitochondria are not only the leading bioenergetic powerhouse, but are also the primary generators of reactive oxygen species (ROS). Normally, there is a dynamic balance between production and neutralization of ROS, and low-level ROS will not produce toxic effects. As a regulator for the development and function of neurons, ROS can help to establish neuronal polarity for growth cone pathfinding and connect the synaptic transmission to the tuning of neuronal networks (Oswald et al., 2018a). Nevertheless, when there is electron leakage, ROS is mainly produced in electron transport chain (ETC) complexes I and III of the mitochondria, thus leading to the generation of superoxide anions (O^2–^) and hydrogen peroxide (H_2_O_2_). ROS dysregulation with the extension of age may interfere with the adaptive adjustment of neurons, thereby resulting in network failure and synapse loss (Oswald et al., 2018b).

Recent evidence has suggested that oxidative stress also causes mitochondrial dysfunction is associated with the development of AD-related pathology (Yu et al., 2018). Due to the limited glycolytic activity of neurons, it is entirely dependent on the energy production of mitochondria. Driven by energy, neurons establish membrane potential, and synthesize, secrete and recycle neurotransmitters, thereby maintaining intracellular Ca^2+^ homeostasis and effectively transmitting neuronal signals. Meanwhile, several studies have shown that mitochondrial respiratory chain and TCA cycle enzymes are significantly reduced (Butterfield et al., 2006). Correspondingly, impaired mitochondrial respiratory chain will further disorder the electron transfer efficiency, increase ROS content, and lead to cellular injury. Also, mtDNA located near the mitochondrial respiratory chain is susceptible to mutations, thus resulting in faulty proteins and ROS (Grimm et al., 2016). In addition, oxidative stress is one of the most prominent hallmarks of the aging process, characterized by the combination of the reduced antioxidant defense system and impaired mitochondrial oxidative phosphorylation (OXPHOS) activity (Grimm and Eckert, 2017). Neurons that rely heavily on OXPHOS to meet their energy demands are particularly prone to energy hypometabolism. Similarly, due to the non-dividing characteristics, neurons could not be replaced in life except that the hippocampus continuously generates new neurons during the adulthood period (Sanchez-Varo et al., 2012). In this case, more and more evidences confirm that energy failure and oxidative stress caused by mitochondrial respiratory dysfunction play a crucial role in the pathogenesis of AD (Marques-Aleixo et al., 2015).

Several clinical evidences have documented that patients with mild cognitive impairment (MCI) have already experienced oxidative stress-induced damage, and this symptom represents the early clinical stage of AD (Aluise et al., 2011). At the same time, the level of biomarkers associated with oxidative stress is directly related to the severity of cognitive impairment and the disease progression from MCI to AD (Ansari and Scheff, 2010). Oxidative stress not only occurs in vulnerable regions of the brain, but also in non-degenerative tissues, such as fibroblasts and lymphocytes, and can precede the Aβ plaque formation and the onset of symptoms of AD. As described, ROS can mainly increase the levels of lipid peroxidation markers such as thiobarbituric acid-reactive substances (TBARS) and 4-hydroxynonenal (4-HNE), thereby accelerating the damage of the brains in AD patients (Butterfield et al., 2002). Similarly, other data have also confirmed that mitochondrial-derived ROS is a sufficient trigger to induce the formation of Aβ (Leuner et al., 2012). In this case, the β-site APP cleaving enzyme 1 (BACE1) activity, which Aβ itself enhances due to the increased ROS level, can accelerate mitochondrial dysfunction and ROS production, thereby enhancing Aβ production in the progression of SAD (Leuner et al., 2012). Furthermore, recent reports have verified that increased production of ROS and susceptibility to the opening of mitochondrial permeability transition pore (mPTP) in APP/PS1/Tau triple transgenic (3xTg) AD model can lead to the increased brain barrier permeability and abnormal brain mitochondria (Carvalho et al., 2013).

Another critical factor for AD pathology is neuroinflammation that is strongly associated with oxidative stress in the brain. For example, Aβ also induces oxidative stress in astrocytes by activating NADPH oxidase, thereby producing chronic neuroinflammation, secreting proinflammatory cytokines, and further exacerbating oxidative stress-induced damage (Mostafa et al., 2016; Wang et al., 2016). At the same time, Aβ can induce increased mitochondrial fragmentation, membrane potential loss and impaired respiratory function of astrocytes (Sarkar et al., 2014). In summary, mitochondrial disorders can be induced by increased oxidative stress and neuroinflammation, thereby contributing to the accumulation of Aβ, and leading to the damage to the structure and function of neurons and synapses as well as astrocytes in the brain tissues, which plays a vital role in the pathogenesis of AD.

### Impaired Mitochondrial Biogenesis in Alzheimer’s Disease

Mitochondrial biogenesis, that is, the growth and division of the pre-existing mitochondria, is a process that requires the coordinated synthesis of proteins encoded by nuclear DNA (nDNA) or mtDNA. Peroxisome proliferator-activated receptor gamma coactivator 1-alpha (PGC-1α) is a central inducer of mitochondrial biogenesis and co-activates the transcription of nuclear respiratory factor 1 (NRF1), which can regulate the transcription of mitochondrial transcription regulator A (TFAM) (Palikaras and Tavernarakis, 2014). When cell energy requirements exceed mitochondrial ATP production, mitochondrial biogenesis is initiated, and mitochondrial regulatory factors will enter the mitochondria to initiate mtDNA replication and transcription and trigger the expansion of the mitochondrial network to meet the demand of the body’s energy metabolism. There is accumulating evidence linking NDs to the defects or mutations of mtDNA. Increasing evidence suggests that an increase in the number of mtDNA mutations in the frontotemporal lobes of the brain in AD patients is consistent with the increase in Aβ42 and hyperphosphorylated Tau in the cerebrospinal fluid, which further confirms the increased mtDNA mutation is associated with the deposition of Aβ in the brain (Podlesniy et al., 2013). When mtDNA is damaged under normal physiological conditions, oxoguanine glycosylase 1 (OGG1) will work with other repairing genes to maintain mtDNA stability. Furthermore, in other clinical studies, the decreased OGG1 activity reveals the early occurrence in the progression of AD, which is possibly mediated by 4-HNE inactivation and may contribute to the elevated 8-hydroxydeoxyguanosine (8-OHdG) in the brain associated with MCI and late-stage AD (Shao et al., 2008).

PGC-1α, the primary regulator of mitochondrial biogenesis, mediates different transcription factors to promote mitochondrial biogenesis. As previously reported, PGC-1α and its significant downstream target genes are down-regulated in the AD brain (Qin et al., 2009) and the M17 cells over-expressing familial AD-causing APP mutant (APPswe) (Sheng et al., 2012), suggesting mitochondrial biogenesis is impaired in AD. PGC-1α is regulated by NRF1 and NRF2, and TFAM encodes, translates and transcribes mitochondrial proteins. Previous studies have found that the expression of PGC-1α, NRF1, NRF2, and TFAM is decreased in cells transfected with APP, and the deposition of Aβ is increased. In parallel, the expression of PGC-1α is negatively correlated with the level of Aβ deposition, indicating that deposition of Aβ can suppress mitochondrial biogenesis (Sheng et al., 2012). Following this line of thought, *in vitro* cultured Tg2576 (an AD mouse model) mouse hippocampal neurons, increased expression of PGC-1α can inhibit the production of Aβ (Porcellotti et al., 2015). Also, the overexpression of TFAM in SH-SY5Y cells can protect them from Aβ-induced mitochondrial dysfunction (Xu et al., 2009), while the overexpression of PGC-1α can effectively inhibit the secretion of Aβ and increase the levels of soluble Aβ in neuroblastoma (N2a) cells (Katsouri et al., 2011). It is also worth noting that the mortality is reportedly increased in APP/PS1 double transgenic mice with specific knockout of SIRT1 that exhibits the reduced activity of SIRT1 deacetylase and increased production of Aβ (Zhang et al., 2011). Further studies have confirmed mitochondrial biogenesis can be promoted by increased SIRT1, which deacetylates and activates PGC-1α to increase its transcriptional activity (Fanibunda et al., 2019). Thus, PGC-1α can regulate mitochondrial biogenesis through the deacetylation of SIRT1 and plays an essential role in the pathogenesis of AD. In addition, Tau also can be deacetylated by SIRT1 (Min et al., 2010), and the level of SIRT1 in the brain is decreased during the progression of AD patients (Julien et al., 2009). Since acetylation inhibits the binding of Tau to microtubules, enhances Tau accumulation by suppressing Tau degradation, and affects the structure and function of neurons in Tau overexpression models such as *C. elegans* single-copy transgenic model (Guha et al., 2020a,b), the activation of SIRT1 can promote the deacetylation of Tau protein, suggesting that targeting SIRT1 activation can serve as an effective strategy for the prevention and mitigation of AD.

### Abnormal Mitochondrial Dynamics in Alzheimer’s Disease

As the highly dynamic organelles, mitochondria present a constant change in shape and status under the regulation of fusion and fission to promote dynamic balance and maintain the health of the mitochondrial network (Mishra and Chan, 2016). On the one hand, the fission/fusion dynamics of mitochondria promote the distribution of mitochondria along the axon to the synapse (Chen et al., 2007), regulate mitochondrial transport and neurotransmitter release, thereby maintaining the transmission of mitochondrial membrane potential (ΔΨm) and calcium buffering (Szabadkai et al., 2006). On the other hand, defective mitochondrial components can be effectively separated during the aging process, thereby preventing damage from ROS (Westermann, 2008). In short, the mitochondrial dynamic is essential for meeting high energy demands and promoting neuroprotection (Chen and Chan, 2009). In the process of regulating mitochondrial dynamics within the neuronal environment, dynamin-related protein 1 (Drp1) and mitochondrial fission protein 1 (Fis1) mainly regulate the process of fission (Su et al., 2010); meanwhile, the fusion process is mediated by mitofusin 1 (Mfn1), mitofusin 2 (Mfn2), and optic atrophy protein 1 (OPA-1) (Youle and van der Bliek, 2012). The morphology and function of mitochondria are regulated dynamically by conserved fission and fusion proteins, and are closely related to cellular homeostasis and aging status (Picard et al., 2013). Additionally, previous studies have also confirmed that mitochondrial fragmentation caused by the dysregulated mitochondrial dynamics, especially the excessive mitochondrial fission, seems to be a vital indicator of neuronal damage (Gao et al., 2017). Another relevant study has shown that abnormal mitochondrial dynamics caused by increased fission is an important early event observed in AD cells (Bonda et al., 2010). In this regard, as previously mentioned, compared with the age-matched control group, the number of mitochondria in neurons of AD mice is reduced, and the proportion of mitochondria with broken cristae is relatively higher (Nixon et al., 2005).

Under normal circumstances, mitochondria dynamically regulate intracellular calcium balance and ATP production, and keep ROS at an appropriate level through continuous fusion and fission to eliminate and repair mutant mtDNA. During the aging process, increased ROS leads to imbalanced mitochondrial homeostasis, and accumulated Aβ and hyperphosphorylated Tau protein cause mitochondrial damage, thereby resulting in pathological changes of AD (García-Escudero et al., 2013). For instance, studies have found that the mitochondrial transition of fission state characterized by increased levels of Drp1 in MCI and AD patients (Silva et al., 2013). Similarly, studies have shown that the increase of Aβ can increase the content of nitric oxide (NO) in the brain and activate the abnormal expression of neuronal Drp1, thus resulting in increased mitochondrial fission and the damage of neurons (Archer, 2013). The most important genetic risk factor for about 90% of SAD is ApoE4. In SAD, ApoE4 can down-regulate the expression levels of the proteins associated with mitochondrial dynamics and ETC (Yin et al., 2020). In the hippocampus of the ApoE4 vector and ApoE4 transgenic mice, the expression of Mfn1, Mfn2, and Drp1 is decreased (Simonovitch et al., 2019). Similarly, the defects in mitochondrial dynamics have been reported in different AD mouse models with Tau overexpression. For example, the expression of P301L mutant Tau can modify mitochondrial dynamics, thereby reducing the expression of both fission and fusion protein regulators (Schulz et al., 2012). In the brain tissues of various AD mouse models with the transfection of APP, APP/PS1, and 3xTg, the binding of Drp1 to hyperphosphorylated Tau protein is enhanced, thereby aggravating mitochondrial dysfunction and synaptic damage (Manczak and Reddy, 2012). Therefore, abnormal mitochondrial dynamics is a contributing factor to AD, and rescuing abnormal mitochondrial dynamics is a valuable therapeutic target for AD.

## The Mitophagy Machinery

Mitophagy refers to the depolarization to mitochondria during starvation, oxidative stress, aging, and ROS damage, and can execute the encapsulation of the damaged mitochondria into the autophagosome, where it fuses with intracellular lysosomes for the degradation of damaged mitochondrial to maintain cellular homeostasis (Fang et al., 2019). Autophagy is categorized into three types: chaperone-mediated autophagy, microautophagy, and macroautophagy. Mitophagy has been identified as a form of macroautophagy that results in selective degradation of dysfunctional or damaged mitochondria, and plays a vital role in the maintenance of cellular function by reducing oxidative stress and restoring cellular homeostasis (Pradeepkiran and Reddy, 2020). This dysfunctional mitochondrial clearance process occurs through the following three forms, namely damage-induced mitophagy, development-induced mitophagy and hypoxia-induced mitophagy (Springer and Macleod, 2016).

### PINK1/Parkin-Mediated Mitophagy

PINK1/Parkin is one of the components in the classic signaling pathway of mitophagy. Under the conditions of stress, injury or aging, mitochondrial serine/threonine kinase PINK1 can sense mitochondrial polarization status, an E3 ubiquitin-protein ligase (Parkin). Under normal conditions, mitochondria have a low membrane potential, and PINK1 is imported into the inner membrane of mitochondria (IMM) and cleaved by mitochondrial-specific protease presenilin-associated rhomboid-like (PARL) (Kane et al., 2014). However, in compromised and depolarized mitochondria, PINK1 is accumulated in the outer membrane of mitochondria (OMM) (Cadete et al., 2019). Mitochondrial protein is phosphorylated by PINK1, thus leading to the recruitment of autophagy cargo adapters and degrading autophagolysosomes by binding to autophagosomes and lysosomes through LC3 (Springer and Macleod, 2016). In order to amplify this signal, PINK1 recruits Parkin from the cytosol by the phosphorylation of Mfn1 and Mfn2. Besides, PINK1 phosphorylates ubiquitin and ubiquitin-like domain (Ubl) of Parkin, which activates Parkin E3 ligase and facilitates its recruitment to OMM (Hamacher-Brady and Brady, 2016). Once in OMM, Parkin can trigger the ubiquitination of Mfn1 and Mfn2, prevent the fusion of mitochondria, and can act as an isolation mechanism when these organelles are damaged, followed by isolation and degradation through selective autophagy (Gomes and Scorrano, 2013). Then, Parkin ubiquitinates OMM protein, such as voltage-dependent anion channel (VDAC1), to generate polyubiquitin chains, recruit autophagy receptor p62, and bind to LC3, thereby degrading and clearing damaged mitochondria (Novak, 2012).

### Bnip3/Nix-Mediated Mitophagy

This type of autophagy driven by Nix/Bnip3, two major pro-apoptotic proteins, is named mitophagy induced by various potential mechanisms (Marinković et al., 2021). Unlike the signal pathway controlled by PINK1/Parkin that cannot directly bind to autophagosome receptors, Bnip3, and Nix can directly bind to autophagy machinery components to mediate mitophagy, including mitochondrial depolarization, PTP aperture, or interference in fission-fusion machinery (Matsuda, 2016). Indeed, the phosphorylation of Bnip3 at Ser17 and Ser24 can promote its binding to LC3-II and Golgi-associated ATPase enhancer of 16 kDa (Gate-16), and facilitate subsequent mitophagy. Recently, Nix has been suggested to induce mitophagy by its interaction with LC3. LC3 interacts with gamma-aminobutyric acid receptor-associated protein (GABARAP) to form the LC3/GABARAP complex, which mediates the mobilization of autophagosomes to mitochondria, for accomplishing the elimination of damaged mitochondria (Hamacher-Brady and Brady, 2016). Besides, Nix can also interact with the Beclin1/Bcl-2 complex to release Beclin1, thus generating the free Beclin1 to induce autophagy (Mishra and Chan, 2016). Furthermore, Bnip3 and Nix are phosphorylated and form homodimers, thereby integrating with OMM and binding to LC3 to induce mitophagy (Zhang and Ney, 2011).

### FUNDC1-Mediated Mitophagy

In addition to classical PINK1/Parkin and Nix/Bnip3-mediated mitophagy, other mitophagy receptors have been discovered, including FUNDC1, and activating molecule in BECN1-regulated autophagy protein 1 (Ambra1) in mammals (Novak, 2012). Under hypoxic conditions, the mitochondrial membrane protein FUNDC1 interacts with LC3 to promote the clearance of mitochondria (Fu et al., 2013). In neurons, the externalization of cardiolipin and its interaction with LC3 has been confirmed to mediate mitophagic degradation of malfunctioning mitochondria (Chu et al., 2013). Additionally, Ambra1, an upstream autophagy regulator of mitochondria, and its pro-autophagy activity are inhibited by Bcl-2 under normal conditions. Upon the induction of mitophagy, Ambra1 binding to LC3 via LC3-interacting region (LIR) motif can increase mitochondrial clearance (Strappazzon et al., 2015). The uncertainty of these results stimulates the necessity for further exploring cellular mechanisms of mitochondrial turnover.

## Dysfunctional Mitophagy and Alzheimer’s Disease

Aging mainly affects mitochondrial homeostasis, and dysfunctional mitochondria could contribute to aging due to their crucial role in the complex balance of cellular processes (Fivenson et al., 2017). Aging-induced ROS generation ultimately causes the mPTP to open and the ΔΨm to depolarize, thus resulting in a rapid impairment of mitochondrial function (Parihar and Brewer, 2007). Moreover, the aging-dependent reduction of mitophagy hinders the elimination of dysfunctional or damaged mitochondria and suppresses mitochondrial biogenesis, thus leading to a progressive accumulation of dysfunctional or damaged mitochondria and the deterioration of cellular function (Kou et al., 2019). Therefore, physiological aging has been associated with the hindrance of mitophagy and impaired mitochondrial function (Figure 2).

As the extension of age, the level of autophagy in neuronal cells is significantly reduced, thus leading to slow mitochondrial renewal, dysfunctional mitochondrial accumulation, cell death, and inflammation, and accelerating senescence, as well as stimulating the development and progression of AD. Moreover, the maintenance of the mitochondrial architecture is critically dependent on the induction of autophagy. The deletion of Atg7 that is required for the formation of autophagosomes can prevent the reestablishment of tubular mitochondria, thereby leading to the obvious accumulation of ROS and cell death (Motori et al., 2013). In addition, autophagy is also essential for regenerating mitochondrial networks in astrocytes. Furthermore, neurons cannot dilute misfolded proteins and damaged organelles through cell fission, so the cellular homeostasis of neurons is highly dependent on autophagy. Mitophagy can promote the elimination of damaged or dysfunctional mitochondria to maintain energy homeostasis, especially for neurons with vital energy requirements (He et al., 2019).

### Effect of Aβ on Mitophagy

There is a complex interplay between Aβ and autophagy, as autophagy controls both the clearance and generation of Aβ. On the one hand, the induction of autophagy can reduce the accumulation of amyloid plaques and rescue neurodegeneration in various systems (Boland et al., 2008). On the other hand, Aβ may also be generated in autophagosomes that appear to contain both the precursor APP and the enzyme presenilin-1 (PS1) responsible for the cleavage of APP to Aβ (Nixon et al., 2005). Furthermore, autophagy is critical for the extracellular secretion of Aβ peptides, as validated by the conditional knockout of the key autophagy-related gene 7 (Atg7), in the APP (Swedish mutation) transgenic mice. However, these events are associated with toxic intracellular accumulation of Aβ, which likely causes neurodegeneration together with amyloidosis and memory deficit (Nilsson et al., 2013).

As a negative regulator of autophagy, mTOR activation will inhibit the initiation of autophagy. The expression of mTOR in the brain of APP/PS1 mice is increased, accompanied by the blocked initiation of autophagy and the increased deposition of Aβ (Wang et al., 2014). However, the injection of mTOR inhibitor, rapamycin, into the brain of APP-mutant AD mouse model can activate autophagy, reduce Aβ pathology, and improve cognitive capacity (Spilman et al., 2010). Besides, the expression of Beclin1 is used as a sign of autophagosome initiation. The expression of Beclin1 in the brain of APP transgenic AD mouse is reduced, and the deposition of Aβ is enhanced (Komatsu et al., 2006). After the initiation of autophagy, the membrane vesicles gradually expand to form the closed autophagosomes. Previous studies have demonstrated that many autophagosomes accumulate in the brain of APP/PS1 mice before Aβ deposits, and many autophagic vesicles containing APP or Aβ accumulate in the brain tissue (Jiang et al., 2014). Consistent with this, a report has described that LC3-II/LC3-I ratio reveals a decrease and p62 expression exhibits an increase in the cortex of AD mouse at the late stage of the disease, and the autophagosomes containing APP and Aβ are gathered in the axon and cannot be transported normally, thus disrupting the fusion of autophagosomes and lysosomes, and leading to the failure of Aβ degradation (Son et al., 2012; Bordi et al., 2016). Alternatively, the excessive accumulation of Aβ can also lead to the destruction of bilayer membrane structure of autophagolysosomes and the abnormal increase of hydrolytic enzymes, thereby causing neuron loss in AD patients (Usenovic and Krainc, 2012). However, the application of lysosomal activators can improve the lysosomal activity in the brain tissues of AD mouse, thereby effectively reducing the deposition of Aβ, and improving learning and memory capacity (Yang et al., 2011).

### Effect of Tau on Mitophagy

Besides Aβ, there is also a link between hyperphosphorylated Tau and mitophagy. Despite being substrates of the ubiquitin-proteasome system, Tau proteins are delivered to the autophagosome-lysosomal system for degradation, so impaired autophagy can lead to the formation of Tau oligomers and the aggregation of Tau insoluble species (Li et al., 2017). Furthermore, autophagy impacts Tau phosphorylation: autophagy-deficient mice display hyperphosphorylated Tau, while autophagy induction reduces Tau phosphorylation (Piras et al., 2016; Uddin et al., 2019). Interestingly, Tau self-modulates the signal pathways of autophagy, as shown in disturbed integrity of lysosome membranes and retarding autophagosome maturation by inhibiting the activity of histone deacetylase 6 (HDAC6) in the presence of Tau aggregation (Uddin et al., 2019). On the other hand, previous studies have reported autophagy in brain cells is declined before the occurrence of AD. Similarly, insufficient Beclin1 in neurons cultured *in vitro* also can cause the accumulation of intracellular Tau hyperphosphorylation. The injection of Beclin1 can reduce the accumulation of hyperphosphorylated Tau protein (Nixon, 2017). In addition, in primary neurons with trehalose, an enhancer of autophagy, the lysosomal activity is significantly increased, and the accumulation of hyperphosphorylated Tau protein is reduced (Krüger et al., 2012).

Genome-wide association studies in AD have identified the variants of phosphatidylinositol-binding clathrin assembly protein (PICALM), which are associated with NFTs and co-localized with conformational abnormalities and hyperphosphorylated Tau proteins in late-onset AD (Ando et al., 2013). PICALM is a clathrin adaptor protein, whose loss-of-function impairs the endocytosis of VAMP2 and VAMP3 (soluble NSF attachment protein receptors-SNAREs involved in the fusion of autophagosome precursors), as well as VAMP8 (involved in the fusion of autophagosomes with lysosomes/late endosomes). Thus, PICALM depletion in transgenic Zebrafish models can inhibit autophagy at multiple steps: from autophagosome biogenesis to maturation and fusion steps, thus ultimately leading to Tau aggregation (Moreau et al., 2014; Menzies et al., 2015). In addition, the hyperphosphorylation of Tau protein also affects mitochondrial function. On the one hand, mitochondrial changes may be secondary to microtubule depolymerization, thus resulting in the inability to transport healthy mitochondria to axons and dendrites, and dysfunctional mitochondria can be removed through autophagy. On the other hand, hyperphosphorylated Tau proteins induce the defects of mitophagy by their inserting into the mitochondrial membrane and impair the mitochondrial residence of PINK1/Parkin (Oliver and Reddy, 2019).

## Protection of Brain Function by Physical Exercise

For a long time, exercise has been validated to be closely correlated with neuronal health in human and rodent animal models. Appropriate exercise has a beneficial effect in slowing down the aging process and enhancing mitochondrial function (Table 1). Similarly, exercise can also enhance the mitochondrial function of peripheral organs to effectively prevent brain atrophy indirectly. At the same time, the beneficial effects of physical activity are now widely accepted on promoting fitness and preventing or treating NDs (Bernardo et al., 2016). Chronic physical activity can stimulate the growth and development of new neural cells in the brain, and plays a vital role in nerve regeneration and neuroprotection. Moreover, exercise can effectively improve the self-protection of cranial nerves, promote the activation of the hippocampal nervous system, and maintain the metabolic activity of the brain (Cass, 2017). Accordingly, exercise can be used as a non-drug interventional strategy to delay or slow down the onset and progression of AD. Nevertheless, the underlying mechanisms of exercise against AD-related mitochondrial dysfunction are not fully understood.

**TABLE 1:** Effect of exercise on oxidative stress, mitochondrial function and autophagy/mitophagy proteins in AD.

Animal model	Exercise training	Oxidative stress	Mitochondrial function	Autophagy/mitophagy	References
TgCRND8 mice	Voluntary wheel running for 6 months	↓Nitro tyrosine		↑Beclin1, STX17	Herring et al., 2016
NSE/hTau23 mice	Treadmill exercise 60 min/day, 5 days/wk for 3 months	↑CuZn-SOD; CAT		↑p-PI3K; p-AKT	Leem et al., 2009
NSE/APPsw mice	Treadmill exercise 30 min/day, 5 days/wks for 8 weeks		↑SIRT1/PGC-1α		Koo et al., 2017
APP/PS1 mice	Treadmill exercise 45 min/day, 5 days/wk for 20 weeks	↑OGG1; 8-OHdG; GPx; ↓Acetyl-MnSOD/MnSOD	↑mtDNA; SIRT3; Complex I/IV;		Bo et al., 2014
3xTg-AD mice	Treadmill exercise 40 min/day, 5 days/wk for 12 weeks		↑p-AMPKα/AMPKα; PGC-1α; NRF1; TFAM; p-Drp1/Drp1; Mfn1; Mfn2	↑LC3-II/LC3-I; Parkin	Kim et al., 2019
3xTg-AD mice	Voluntary wheel running for 3 months	↑CuZn SOD; ↓LPO; MnSOD; GR; GPx; GSSH			García-Mesa et al., 2016
3xTg-AD mice	Voluntary wheel running for 6 months	↑GPx; MnSOD; GSH; ↓LPO	↑mtDNA		García-Mesa et al., 2012
SAMP8 mice	Voluntary wheel running, 3 days/wk for 25 weeks		↑AMPK/SIRT1/PGC-1α; Complex I-V;		Bayod et al., 2015
C57BL/6J mice	Treadmill exercise 60 min/day, 5 days/wk for 3 weeks and 2 days		↑Complex I/III; Drp1;		Gusdon et al., 2017
STZ-induced AD model rats	Treadmill exercise 30 min/day, 5 days/wk for 4 wks	↓3-NT; 4-HNE; p-H2AX; 8-OHdG	↑CCO; mtDNA		Lu et al., 2017
Wistar rat	Treadmill exercise 45 min/day, 5 days/wk for 4 weeks		↑AMPK/PGC-1α		Azimi et al., 2018
Sprague Dawley rat	Swimming training 30 min/day, 5 days/wk for 10 weeks	↑SOD-1; TBARS; 4-HNE; nitrotyrosine	↑mtDNA; PGC-1α; Mfn2; Drp1; Complex I/IV;	↑Atg5; Parkin ↓LC3-II/LC3-I; p62	Luo et al., 2017
Sprague Dawley rat	Treadmill training 60 min/day, 5 days/wk for 12 weeks Voluntary wheel running	↑Mn-SOD; aconitase; UCP2 ↓MDA; p66shc(pSer36)/p66shc;	↑PGC1α; TFAM; SIRT3; Mfn1/2; Complex I/V ↓Drp1	↑LC3-II/LC3-I; Beclin1; PINK1	Marques-Aleixo et al., 2015
Sprague Dawley rat	Swimming training 90 min/day, once a day for 6 weeks	↑SOD ↓MDA	↑PGC-1α; ↓Drp1; Mfn2	↑LC3-II/LC3-I; Beclin1; Atg7;↓p62	Kou et al., 2017
MCI patients	Treadmill/resistance exercise, 40 min/day, 3 days/wk for 16 weeks	↓TNF-α; IL-15	↑BDNF	↑IGF-1	Tsai et al., 2019
AD patients	Treadmill/stationary bike/cross trainer exercise, 60 min/day, 3 days/wk for 16 weeks	↓IL6			Jensen et al., 2019

*3-NT, 3-nitrotyrosine; 4-HNE, 4-hydroxynonenal; p-H2AX, DNA double-strand breaks; 8-OHdG, 8-hydroxydeoxyguanosine; CCO, cytochrome C oxidase; Cu/Zn-SOD, copper/zinc superoxide dismutase; CAT, catalase; LPO, lipoperoxide; SOD, manganese superoxide dismutase; GR, glutathione reductase; GPx, glutathione peroxidase; GSH, glutathione; GSSG, glutathione disulfide; STX17, syntaxin 17; OGG1, oxoguanine glycosylase 1; SOD, superoxide dismutase; TBARS, thiobarbituric acid reactive substances; MDA, malondialdehyde; UCP2, uncoupling protein 2; wk (s), week (s); min, minutes; ↑significant increase; ↓significant decrease.*

### Exercise Reduces Oxidative Stress

To date, approximately 30% of adults worldwide are underactive, which becomes a more significant risk factor for abnormal ROS. At the same time, the biomarkers associated with low antioxidant capacity and high-level oxidative stress can be determined in AD patients at the early stage (Schrag et al., 2013; Brown et al., 2017). It has been confirmed in different biological models that exercise is closely correlated with improved antioxidant capacity of the brain and reduced oxidative stress-induced damage, thereby preventing redox changes associated with aging and NDs. In addition, 4-week treadmill exercise can significantly promote the neurogenesis of streptozotocin (STZ)-induced AD model rats, accompanying with reduced 4-HNE, DNA double-strand breaks (p-H2AX), and 8-OHdG, as well as reduced Aβ aggregation and hyperphosphorylated Tau protein in the brain (Lu et al., 2017). Similarly, in NSE/hTau23 mice following 3 months of treadmill exercise, the increased activity of antioxidant enzymes such as SOD and CAT in the brain is detected, while the expression of hyperphosphorylated Tau protein is decreased (Leem et al., 2009). Another previous study through 3xTg-AD mice for 3-month voluntary wheel running has documented that exercise can significantly reduce Aβ deposition and Tau hyperphosphorylation, reduce oxidative indicators such as glutathione disulfide (GSSG), and improve SOD activity in the hippocampal tissue, suggesting that exercise can improve redox status, reduce Aβ accumulation and Tau hyperphosphorylation and enhance brain memory function (García-Mesa et al., 2016). However, exercise regimens with different intensities, durations and loads can lead to distinct physiological and functional adaptation of the brain. Moreover, age and animal characteristics also affect the variability of experiments, which may explain the controversial results between oxidative stress in the brain and physical exercise. For example, some studies have found that endurance training can decrease lipid peroxidation and oxidative DNA damage, while acute exercise cannot cause any significant alteration of mitochondria in the brain (Liu et al., 2000). The above findings indicate that mitochondrial adaptation may be affected by the bioenergetic properties of exercise.

In addition, moderate exercise combined with antioxidant supplementation can synergistically reduce the age-dependent risk of protein and lipid oxidation modification in the brain, thereby significantly activating the endogenous antioxidant system (Sakr et al., 2015). In the study of AD mouse models, moderate exercise can inhibit the elevated level of oxidative stress in the brain and maintain redox homeostasis (Kim et al., 2019). ROS generation during exercise may play an important role in regulating mediator molecules to affect adaptive responses. ROS also can modulate the expression of antioxidant proteins and uncoupling proteins (U) (Andrews et al., 2005), as well as heat shock proteins (HSPs) (Calabrese et al., 2000). This combinatorial up-regulation mediated by feedback mechanisms can prevent the extent of oxidative stress and apoptosis, ultimately promoting neuroprotection. Overall, exercise training can be used as a potentially effective strategy for enhancing antioxidant enzyme activity of neurons, reducing the release of mitochondrial ROS, improving the excessive oxidation of lipids, proteins and DNA, and reducing the level of oxidative stress and apoptosis with subsequent attenuation of AD progression.

### Exercise Enhances Mitochondrial Biogenesis

During exercise, energy requirements reveal a sharp increase, and mitochondria can increase the source of available energy. Mitochondrial biogenesis is the basis for maintaining the number of mitochondria and a guarantee for meeting the needs of energy metabolism in neurons. Damaged mtDNA is one of the critical factors for leading to abnormal mitochondrial biogenesis in the AD brain. Recent studies have confirmed that l6-month treadmill exercise can stimulate a significant increase in the expression of oxidative factors such as GSSG, and an improved metabolic level of neurotransmitters such as acetylcholine, glutamate, and aspartate in the brain of 3-month-old mtDNA mutant mice (Clark-Matott et al., 2015). Similarly, another study has reported that the combinatorial therapy with metformin and 6-week treadmill exercise can significantly reduce Aβ accumulation and Tau hyperphosphorylation, up-regulate the activities of SOD and CAT, and down-regulate damaged mtDNA in the brain of the mice, thereby improving learning and memory capacity (García-Mesa et al., 2012). Recently, an animal model study has found that 20-week treadmill exercise can significantly increase manganese superoxide dismutase (MnSOD), ATP production and OGG1, with a significant decrease in Aβ deposition and ROS production in the brain of APP/PS1 mice, suggesting that exercise can reduce mtDNA damage and increase the amount of mtDNA by increasing the activity of mtDNA-repairing enzymes (Bo et al., 2014).

The dynamic balance between mitochondrial biogenesis and mitochondrial clearance and renewal is critical in maintaining mitochondrial function in neurons. Exercise can stimulate mitochondrial biogenesis and maintain the integrity of neuronal structure and function. Recent studies have confirmed that, in 6-month-old SAMP8 female rapidly aging mice, 2-week voluntary wheel running can promote mitochondrial biogenesis by increased SIRT1 and OXPHOS substrates in the hippocampal tissues of mice (Bayod et al., 2015). Similarly, 8-week exercise can decrease Aβ deposition via the SIRT1/PGC-1α signaling pathway, thereby improving learning, and memory capacity (Koo et al., 2017). Furthermore, the mice subjected to 4 weeks of treadmill training exhibit higher learning and memory capacity, higher expression levels of AMPK, PGC-1α and other regulatory factors of mitochondrial biogenesis, and lower levels of Aβ deposition in the brain of AD rats (Azimi et al., 2018). Collectively, exercise can improve the brain function by increasing mitochondrial biogenesis, improving mitochondrial metabolic function and reducing neuronal Aβ deposition via the AMPK/SIRT1/PGC-1α signaling pathway in the brain of AD subjects.

### Exercise Improves Mitochondrial Dynamics

In order to stabilize the normal status of cells, mitochondria urgently need to reorganize through fusion and fission cycles so that damaged or dysfunctional mitochondria can be specifically repaired or eliminated, thereby maintaining the integrity of mitochondrial structure and function. According to a previous report, resistance exercise has almost no effect on mitochondrial biogenesis, but it can change the activation status and total amount of mitochondrial-related fusion/fission proteins (Kitaoka et al., 2015). Additionally, in comparison with untrained mice, the mice subjected to swimming training exhibit the better cognitive function, stronger rejuvenated mitochondria, higher expression levels of PGC-1α, Mfn1/2, and Drp1 proteins, and lower level of oxidative stress (Luo et al., 2017). Similarly, according to current studies, swimming training can significantly improve the learning and memory capacity of D-galactose-induced AD model rats, and induce autophagy by suppressing miR-34a, as well as promote the expression of Mfn2 to improve mitochondrial quality control (Kou et al., 2017). A recent study has reported 12-week treadmill exercise and voluntary wheel running can increase mitochondrial fusion proteins such as Mfn1/2, and suppress the fission-related proteins such as Drp1 to increase mitochondrial plasticity in the cortex and cerebellum of the brain (Marques-Aleixo et al., 2015).

However, another study has demonstrated exercise training can improve mitochondrial function in the brain by improving the function of ETC and promoting a shift in the mitochondrial fission-fusion balance toward fission (increased Drp1) in aged mice (Gusdon et al., 2017). As brain energetic stress can induce mitochondrial fission arrest in AD, the up-regulation of Drp1 may reverse aging-induced mitochondria-on-a-string (MOAS) formation and preserve residual mitochondrial function in the brain (Zhang et al., 2016). Thus, all above results have confirmed that exercise can enhance the dynamic balance of mitochondrial fusion and fission, and maintain brain health.

### Exercise Stimulates Mitophagy in Alzheimer’s Disease

Under normal physiological conditions, mitochondria eventually split into two uneven offspring. Mitophagy can selectively reduce the membrane potential and degrade mitochondrial progeny that exceeds their repairing capability. Usually, mitochondria responding to depolarization under stress stimuli (such as ROS, nutrition deficiency and exercise), are wrapped into autophagosomes and fused with lysosomes to degrade the damaged mitochondria and misfolded proteins (such as Aβ, Tau protein) in the brain through mitophagy as a form of selective autophagy, thereby maintaining mitochondrial metabolism in the brain. Exercise-induced autophagy caused by treadmill running can improve the learning and memory capacity, which is first discovered in the cerebral cortex of normal mice in 2012 (He et al., 2012).

Currently, increasing evidence confirms the beneficial effects of different exercise regimens on the functional status of autophagy in brain tissues of rodent models. The mice subjected to treadmill running and voluntary wheel running exhibit a higher expression level of LC3-II protein, and a lower expression level of p62 protein in the cerebral cortex of aged mice (Rocchi and He, 2017). The latest study has demonstrated 12-week voluntary wheel running and treadmill exercise can also increase the expression of Beclin1 and LC3-II, and reduce the expression of p62 in the brain of Sprague-Dawley (SD) rats, suggesting that both active and passive exercise can promote the formation of autophagosomes, and accelerate the degradation of damaged organelles and mis-folded proteins by autolysosomes to maintain normal brain functions (Marques-Aleixo et al., 2015).

Similar findings have been reported that 10-week swimming exercise in young SD rats can significantly up-regulate the expression of mitophagy biomarkers LC3-II/LC3-I ratio and Parkin, while the expression of p62 is decreased in the brain of rats, further confirming that exercise can activate mitophagy (Luo et al., 2017). However, the mechanism by which exercise mode achieves this effect needs to be further explored. Furthermore, another study has verified that voluntary wheel running for 5 months can increase the expression of Beclin1 and Atg5 in the cerebral cortex and hippocampus of both 7- and 1-month-old TgCRND8 (expressing a mutant human APP695 gene) mice, thereby suppressing the Aβ deposition in the brain of AD mice (Herring et al., 2016). These studies suggest that exercise can increase the level of mitophagy at different stages of AD, accelerate the clearance rate of Aβ in the manner of mitophagy, and play a certain role in preventing and relieving AD. Additionally, numerous studies have demonstrated that exercise activates the PI3K/Akt/mTOR signaling pathway to increase synaptic plasticity, control neuron survival, suppress apoptotic cell death, and improve the learning and memory capacity of mice (Chae and Kim, 2009; Fang et al., 2013). Further studies on exercise-induced autophagy/mitophagy will be required to assess exercise as a critical built-in mechanism for promoting mitochondrial renewal cycle and functional improvement in AD subjects.

## Perspectives for Future Studies

**FIGURE 3:**
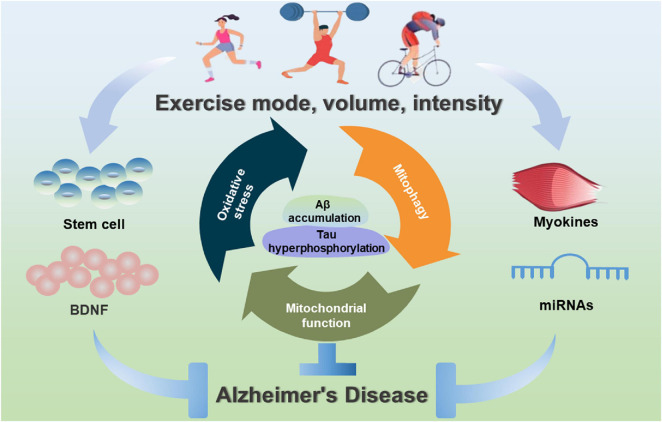
Exercise-induced benefits of mitochondrial-targeted therapeutics for AD. Exercise interventions can contribute to mitochondrial health, inhibit oxidative stress, improve mitochondrial biogenesis and mitochondrial dynamics, and induce mitophagy, and stimulate myokines, miRNAs and stem cells to improve mitochondrial function during the prevention and treatment of AD.

This article focuses on the critical benefits of physical activity in the prevention and treatment of AD. Interestingly, the specific type, intensity and duration of exercise are very different in reducing the risk of AD or delaying the onset of AD. To date, exercise training can effectively alleviate or rescue Aβ and Tau pathologies, reduce neuroinflammation, stimulate hippocampal neurogenesis and improve cognitive function of AD. In terms of exercise time to prevent and treat AD, cognitive improvement can be observed after 3 weeks; however, the decrease in Aβ amount is only observed after 10 weeks of running in aged Tg2576 mice (Parachikova et al., 2008). Besides, a decrease in hyperphosphorylated Tau protein and an improvement in spontaneous alternation are also observed after 9-month voluntary running (Tapia-Rojas et al., 2016). However, the intensity of exercise training must be considered, especially in long-term exercise training. Among them, low-intensity training can increase cell proliferation and neurogenesis, and prevent excessive activation of the hypothalamic-pituitary axis (Naylor et al., 2005). Vigorous exercise is a bioenergetic challenge that can enhance the capacity of neurons to stress resistance (Mattson, 2015). Interestingly, high-intensity exercise with a high load has adverse effects. Previous studies have demonstrated that appropriate low-intensity exercise is more effective than high-intensity exercise in protecting and restoring the aging brain in rats (Kim et al., 2003). In clinical trial cases, the results present a wide variation depending on the type and intensity of exercise (Enette et al., 2020). Thus, there are still many questions about the potential benefits of exercise in AD animal models and human patients. Although, the literature reviewed in this article has consistently proved the promotion of exercise on learning and cognitive enhancement and reduced pathological amyloid (Figure 3). However, pre-clinical studies still do not have clear recommendations to define the best exercise type, intensity, and duration. In the future cohort studies, it should be stratified according to disease severity and use combinatorial interventions with appropriate exercise prescriptions for patients with AD.

Although the brain is a non-contracting tissue, increased metabolism can indirectly affect neuronal function. The cross-talk of the brain and skeletal muscle may explain how physical exercise improves brain function. Changes in the content and activity of antioxidant enzymes in skeletal muscle induced by exercise can limit the applications of glucose and have indirect impacts on the brain metabolism, thus protecting the increased metabolic needs of the brain during and after exercise (Pilegaard et al., 2000). During the contractile activity, the secretome contains hundreds of myokines contributing to the adaptation of various organs in a paracrine or endocrine fashion (Laurens et al., 2020). Exercise also affects body fat, metabolic activity, and functions of heart and skeletal muscle. However, how does exercise promote the health of mitochondria in the brain (or other tissues)? The combination of peptides, lipids, messenger RNAs (mRNAs), microRNAs (miRNAs), and mtDNA released during muscle contraction are collectively referred to as myokines (Trovato et al., 2019; Kou et al., 2020). As one of these myokines, irisin can potentially mediate signaling between skeletal muscle and brain, thereby transmitting the beneficial effects of exercise to the brain (Lu et al., 2016). Numerous studies have supported mitochondrial dysfunction in the central nervous system and peripheral tissues from AD patients (Atamna and Kumar, 2010; Silzer et al., 2019). More and more clinical trials have validated that exercise can increase neurotrophic factors, reduce inflammatory cytokines, and promote neurocognitive performance both in healthy elderly (Mejías-Peña et al., 2017) and AD patients (Jensen et al., 2019; Tsai et al., 2019). However, due to limited clinical studies on the role of exercise-regulated mitochondria in the pathogenesis of AD, and it is worthwhile to elucidate the molecular effects of exercise in AD.

At the same time, more recent evidence shows that stem cells play a neurotrophic role after transplantation and increase the levels of a variety of neurological factors, such as BDNF that plays a paracrine role to improve various cellular functions in animal models with AD, including synaptic strength, neurogenesis, mitochondrial function, autophagy and apoptosis (Fang et al., 2018). Therefore, compared with the conventional treatment of a single pathology, exercise combined with multiple treatments appears to be more promising in the prevention and treatment of AD.

## Conclusion

Maintaining the integrity of mitochondria is an essential factor in promoting the practical function of cells. Any abnormal mitochondrial functions may adversely affect cells such as neurons that are critically dependent on mitochondria. Mitochondrial dysfunction, defects in mitochondrial dynamics and impaired mitophagy can lead to increased oxidative stress, synaptic dysfunction, neuronal loss and cognitive impairment, thereby inducing the development and progression of AD. Although the considerable improvement in the management of AD is achieved, novel and effective pharmacological interventions have not been determined. As an effective non-drug interventional strategy, exercise is beneficial to alleviate or delay the occurrence and development of AD. Appropriate exercise can enhance mitochondrial quality control, such as promoted mitochondrial biosynthesis and accelerated degradation of damaged or aging mitochondria. Meanwhile, appropriate exercise also can enhance the efficiency of mitochondrial dynamics and activate mitophagy, thereby reconstructing the optimal mitochondrial network, improving the plasticity of nerves, and providing practical strategies for healthy aging and the prevention of AD.

## Author Contributions

JL and CW prepared the first draft and final version of the manuscript. HZ and JH were involved in literature searching. JX and NC critically edited and revised the manuscript. All authors have read and approved the final version of the manuscript.

## Conflict of Interest

The authors declare that the research was conducted in the absence of any commercial or financial relationships that could be construed as a potential conflict of interest.

## Publisher’s Note

All claims expressed in this article are solely those of the authors and do not necessarily represent those of their affiliated organizations, or those of the publisher, the editors and the reviewers. Any product that may be evaluated in this article, or claim that may be made by its manufacturer, is not guaranteed or endorsed by the publisher.
